# Mast cell heparanase promotes breast cancer stem-like features via MUC1/estrogen receptor axis

**DOI:** 10.1038/s41419-024-07092-9

**Published:** 2024-09-30

**Authors:** Roberta Bongiorno, Mara Lecchi, Laura Botti, Oriana Bosco, Chiara Ratti, Enrico Fontanella, Nicolò Mercurio, Pietro Pratesi, Claudia Chiodoni, Paolo Verderio, Mario Paolo Colombo, Daniele Lecis

**Affiliations:** 1 Molecular Immunology Unit, Department of Experimental Oncology, Fondazione IRCCS Istituto Nazionale dei Tumori di Milano, Milano, MI Italy; 2 Bioinformatics and Biostatistics Unit, Department of Epidemiology and Data Science, Fondazione IRCCS Istituto Nazionale dei Tumori di Milano, Milano, MI Italy

**Keywords:** Cell biology, Breast cancer

## Abstract

Breast cancer is the most frequent type of tumor in women and is characterized by variable outcomes due to its heterogeneity and the presence of many cancer cell-autonomous and –non-autonomous factors. A major determinant of breast cancer aggressiveness is represented by immune infiltration, which can support tumor development. In our work, we studied the role of mast cells in breast cancer and identified a novel activity in promoting the tumor-initiating properties of cancer cells. Mast cells are known to affect breast cancer prognosis, but show different effects according to the diverse subtypes. Starting from the observation that co-injection of mast cells with limiting concentrations of cancer cells increased their in vivo engraftment rate, we characterized the molecular mechanisms by which mast cells promote the tumor stem-like features. We provide evidence that mast cell heparanase plays a pivotal role since both its activity and the stimulation of mast cells with heparan sulfate, the product of heparanase activity, are crucial for this process. Moreover, the pharmacological inhibition of heparanase prevents the function of mast cells. Our data show that soluble factors released by mast cells favor the expression of estrogen receptor in a MUC1-dependent manner. The MUC1/estrogen receptor axis is eventually essential for cancer stem-like features, specifically in HER2-negative cells, and promotes the capability of cancer cells to form mammospheres and express stem-related genes, also reducing their sensitivity to tamoxifen administration. Altogether our findings describe a novel mechanism by which mast cells could increase the aggressiveness of breast cancer uncovering a molecular mechanism displaying differences based on the specific breast cancer subtype.

## Introduction

Breast cancer is the most frequent tumor in women and is responsible for about 45000 deaths per year in the USA [1]. It is a very heterogeneous disease due to both cancer cell-intrinsic and -extrinsic determinants. Cancer cell phenotype depends on the tissue of origin of the tumor, the presence of mutations, and it is also determined by the influence of tumor microenvironment (TME). The latter is constituted by stromal cells, extracellular matrix (ECM) and immune cells, which can hinder cancer progression [2], but can also be supportive of tumors [3]. Breast cancer is usually classified according to the expression of estrogen and HER2 expression [4], and we have recently shown that the activity of these receptors can be modulated by the presence of mast cells [5].

Mast cells are c-Kit-positive immune cells which usually localize in mucosa and skin [6]. They are considered sensors of microenvironmental changes and are able to trigger very rapid and strong responses, which are evident, for example, in allergic reactions. Mast cells are also studied in oncology and have been shown to affect prognosis in several types of tumors [7]. Interestingly, via single-cell analysis [8] or by exploiting mast cell-related signatures derived by gene expression profiles of tumors [9, 10], mast cells have been shown to either promote cancer progression [11, 12] or display an antitumor effect depending on tumor types [13]. Many mast cell activities could contribute to this opposite effect in tumors. Besides their immunological functions and as modulators of other immune populations [14, 15], mast cells can also affect cancer cell features, for example, by preventing a basal phenotype, as we have recently demonstrated [5]. By modulating the activity of estrogen receptor, mast cells can also influence the biology and progression of breast cancer due to the crucial role of hormone regulation in this type of tumor [16]. Notably, mast cells are not the only immune cells known to impact on the phenotype and behavior of cancer cells. Failure of cytotoxic activity of T cells was shown to promote cancer cell stem-like features in breast cancer [17]. Moreover, tumor-infiltrating immune cells, such as macrophages, can promote epithelial-to-mesenchymal features in adjacent cancer cells [18]. Interestingly, also macrophages stimulate estrogen receptor, as shown for mast cells, and this effect has been hypothesized to depend on heparanase activity [19].

Heparanase modulates the structural and biochemical functions of heparan sulfate proteoglycans through the degradation of heparan sulfate. Heparanase is considered a therapeutic target in anti-cancer therapy [20] since it contributes to several processes, which are crucial for cancer progression, such as proliferation, inflammation, extracellular matrix shaping, angiogenesis, resistance to therapy, and dissemination by metastasis [21]. Moreover, via proteoglycan degradation, heparanase promotes the release and activity of chemokines and cytokines. In our present work, we show that mast cells support tumor-initiating properties of cancer cells, and provide evidence that mast cell heparanase is crucial for this effect. In fact, we demonstrate that the conditioned medium collected from mast cells is able to increase the capability of cancer cells to form mammospheres in a MUC-1/estrogen receptor-dependent manner, and the whole mechanism can be prevented by inhibiting heparanase. Finally, we also identified TNF as a player of this mast cell-dependent process.

## Results

### Mast cells increase the tumor-initiating properties of breast cancer cells via induction of stem-like features

**Fig. 1:**
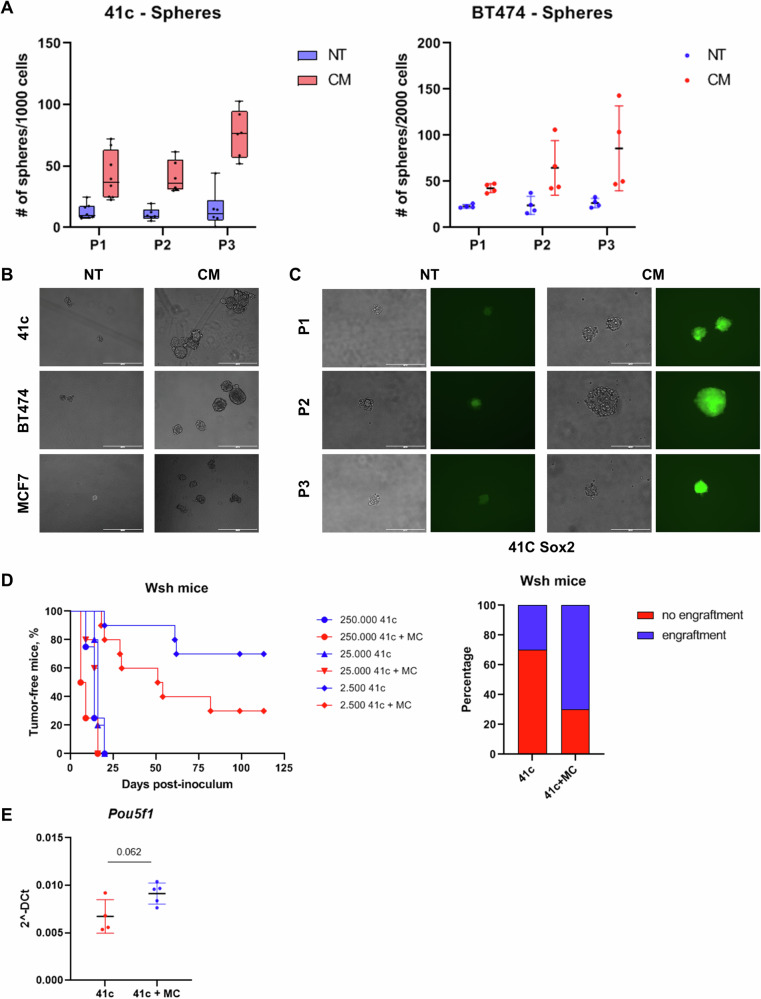
Mast cells enhance the tumor-initiating potential of cancer cells. **A** Mouse (left) and human (right) cancer cell lines were tested in mammosphere-forming assay in the presence of normal (NT) and mast cell conditioned (CM) medium. **B** Representative images of spheres obtained in mammosphere assays performed with mouse (PyMT41c) and human (BT474 and MCF7) breast cancer cell lines. Scale bars = 400 µm. **C** PyMT41c cells were transduced with a GFP-based Sox2 promoter-reporter and cultured in mammosphere-forming assay. Representative bright field and fluorescent images of cells cultured in normal (NT) and mast cell conditioned (CM) medium were taken at P1, P2 and P3. Scale bars = 400 µm. **D** PyMT41c cells were injected alone at different doses (250.000 *n* = 4, 25.000 *n* = 5 and 2500 *n* = 10) or coinjected with mast cells (1:1) in the fat pad of Wsh mice. At the limiting dose of 2.5 × 10^3^ cells/mouse (*n* = 20), the difference between proportions of engrafted in the two groups was equal to Δp=0.40 with a 95% Confidence interval (-0.002; 0.802) and a *p*-value: 0.074. **E** Ex vivo analysis of Pou5f1 mRNA levels expressed by tumors grown in the presence of mast cells (41c + MC, *n* = 5) or not (41c, *n* = 4), Wilcoxon’s *p*-value.

In our previous work, we observed that co-injection of a mouse mammary cancer cell line with mast cells resulted in a faster engraftment of syngeneic mice [5]. We hence hypothesized that mast cells could increase the tumor-initiating capability of mammary cancer cells and verified this assumption both in vitro and in vivo. First, cancer cell lines were tested in mammosphere forming assay to determine whether mast cells are able to increase their capability to form spheres (Fig. 1A-C). Cancer cells were cultured in the presence of a medium conditioned by mast cells for 24 h, finding that the conditioned medium was sufficient to significantly increase the number of spheres for at least three passages. Both mouse-derived (PyMT41c) and human commercial (BT474 and MCF7) cancer cell lines formed more spheres in the presence of conditioned medium (Figs. 1A and B). In the PyMT41c experiment, a different effect of the passage was observed between the NT or CM groups (interaction p-value: 0.042), whereas a persistent significant CM’s effect was observed in each passage (p-value CM in P1: 0.002, *p* value CM in P2: 0.001, p-value CM in P3: 0.001). In the BT474 experiment, CM’s effect resulted statistically significant (CM *p* value: <0.001; Passage *p* value:0.311). Moreover, to detect the expression of stem-related genes, PyMT41c cells were transduced with a GFP-based Sox2 reporter to investigate the activity of the Sox2 promoter, which was indeed increased by the presence of conditioned medium (Fig. 1C).

Based on these findings, we then tested the capability of mast cells to increase the tumor-initiating capacity of cancer cells in vivo. PyMT41c cells were injected at scaling doses with or without mast cells in Wsh mice, which are deficient for endogenous mast cells. At the higher doses tested (2.5x10^5^ and 2.5x10^4^ cells/mice), PyMT41c cells engrafted 100% of mice. At the limiting dose of 2.5×10^3^ cells/mouse, a small difference was observed in tumor volumes with or without mast cells, but, at the end of the experiments, 70% of mice co-injected with mast cells developed tumors, while only 30% of mice injected with tumor cells alone presented palpable tumors (Fig. 1D). Interestingly, ex vivo analysis of tumors showed that tumors grown in the presence of mast cells express higher expression of Oct4/Pou5f1 (Fig. 1E), while Sox2 and Nanog stem-related factors were not affected by MC co-injection (Supplementary Figure 1). Altogether, these results support the idea that mast cells can increase the capability of mammary cancer cells to initiate tumors by increasing their stem-like properties.

### Heparanase contributes to mast cell capability to stimulate cancer stem-like traits

**Fig. 2:**
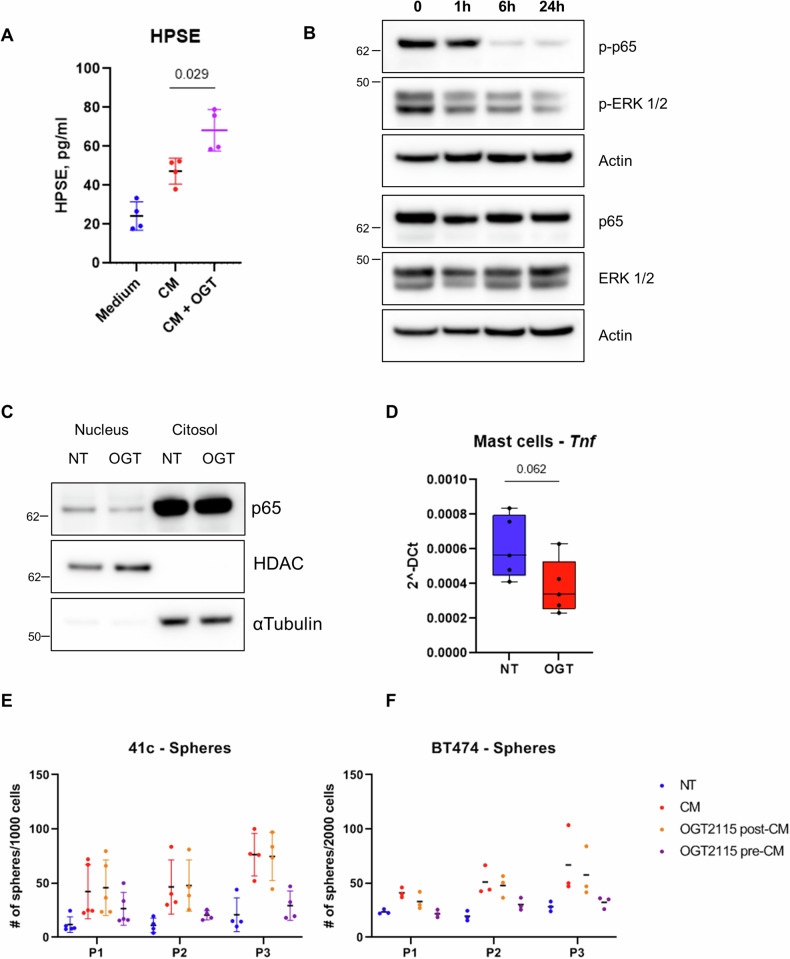
Mast cell heparanase is involved in the induction of stem-like features. **A** Mast cell conditioned medium (CM) was analyzed by ELISA to assess the presence of heparanase secreted by mast cells untreated and treated with OGT2115, Kruskal Wallis’s p-value: <0.001. Wilcoxon’s p-value is shown in the graph. **B** Mast cell protein lysates were collected after treatment with heparanase inhibitor OGT2115 (2 µM) for the indicated time, and tested by western blot to evaluate the levels of phosphorylated and total p65, and ERK 1/2. Actin is shown as loading control. **C** nuclear/cytoplasm protein extraction of mast cells treated for 24 hours with OGT2115 were analyzed by western blot to detect p65 localization. HDAC is shown as nuclear and αTubulin as cytoplasmatic markers. **D** Real-Time PCR performed on mast cells treated as in panel **B** to detect *Tnf* expression levels. Friedman’s p-value is indicated. **E**, **F** PyMT41c and BT474 cells were tested in mammosphere forming assay in the presence of normal (NT), mast cell conditioned (CM) medium, or medium conditioned by mast cells treated with OGT2115 for 24 hours before the collection of the medium (OGT2115 pre-CM). OGT2115 was added also in the conditioned medium after its collection to treat cancer cells directly (OGT2115 post-CM).

We have previously shown that mast cells can stimulate the expression and activity of estrogen receptor in adjacent breast cancer cells. A similar effect has been reported also for macrophages [19] where authors demonstrated that a crucial role in this process is played by heparanase. We hence asked whether this enzyme is responsible for the stimulation of estrogen receptor also in our models and whether it playes a role in the pro-tumor-initiating effect observed. We first confirmed that also mast cells secrete heparanase in the medium (Fig. 2A) and that the pharmacological inhibition of this enzyme (with OGT2115) reduced the activation of ERK1/2 and p65/NF-kB (Fig. 2B). These pathways have been shown to be positively regulated by heparanase [22–24] and are constitutively active in our culturing conditions (Fig. 2B). Western blot confirmed that the concentration of OGT2115 used was able to inhibit ERK1/2 and NF-kB activity, supporting the idea that heparanase controls these pathways also in mast cells, likely affecting their activity. Moreover, OGT2115 reduced the nuclear translocation of p65 (Fig. 2C) and the expression of the NF-kB target gene *Tnf* (Fig. 2D). Notably, the administration of OGT2115 prevented the activation of heparanase down-stream pathways without reducing heparanase protein levels, which were actually increased (Fig. 2A).

To determine the effect of heparanase in the tumor-initiating properties described above, we added the heparanase inhibitor to mast cells before collecting the conditioned medium (pre-). This medium was then employed in mammosphere forming assays which revealed that the inhibitor reduced the number of spheres compared to conditioned medium (Fig. 2E, F). We then asked whether heparanase itself was directly responsible for enhanced mammospheres-forming capacity of cancer cells and added the heparanase inhibitor directly to cancer cells. In this case, the same concentration of inhibitor used to treat mast cells was added to the conditioned medium after being collected (post-). Notably, direct treatment of cancer cells with OGT2115 had no effect on mammosphere formation, suggesting that cancer cell heparanase is not directly responsible for this effect, but it plays an autocrine role via mast cell activation/stimulation. Findings were confirmed by using both mouse (PyMT41c, Fig. 2E) and human (BT474, Fig. 2F) mammary cancer cells. In both experiments, there is a significant group’s effect (Group *p* value: <0.001), with contrasts CM vs. OGT2115 pre-CM or OGT2115 post-CM vs. OGT2115 pre-CM statistically significant (PyMT41c contrasts *p* value: 0.002 and 0.001; BT474 contrasts *p* value: <0.001 and 0.001, respectively). These findings support the notion that the capability of mast cell heparanase to promote the stem-like features is broadly valid and not cell-line specific.

### Mast cells promote estrogen receptor expression and stem-like features via MUC1 and heparanase

Since we had previously demonstrated that mast cells trigger estrogen receptor expression in breast cancer cells [5] and we show here that they also promote their stem-like features, we looked for molecules, which could be involved in both mechanisms. We focused on MUC1, a protein which has been shown to favor estrogen receptor transcriptional and post-translational [25] expression, and to regulate cellular stem-like properties [26]. MUC1 expression is stimulated by inflammation [27] and we hence hypothesized that mast cells could be responsible for its upregulation in cancer cells.

**Fig. 3:**
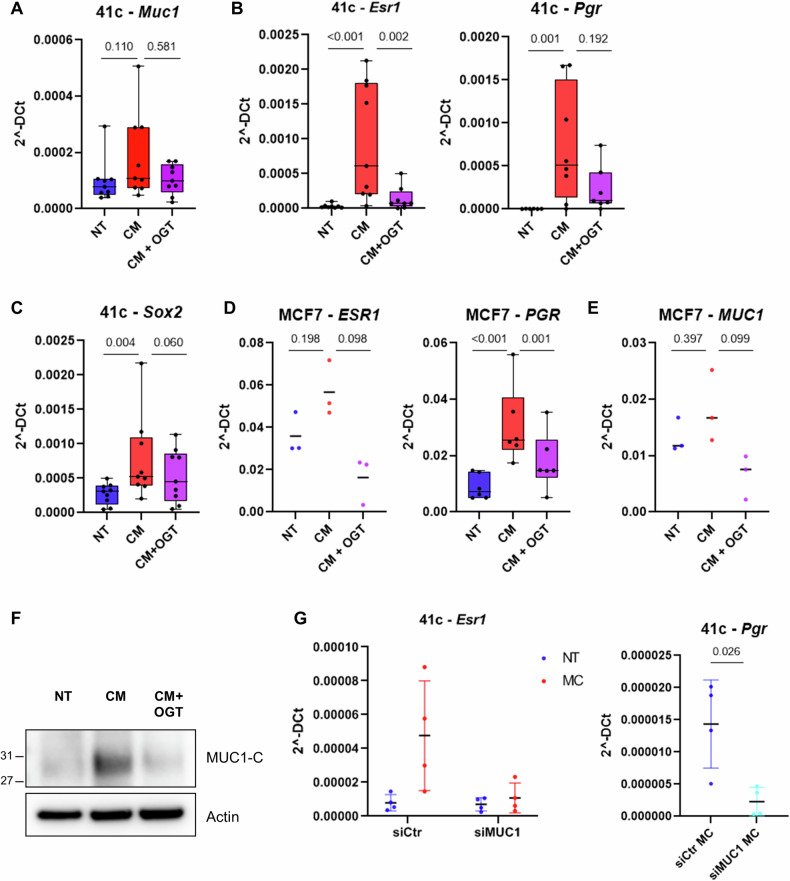
Mast cells induce estrogen receptor and Sox2 expression in a heparanase/MUC1-dependent manner. PyMT41c cells were cultured for 24 hours in normal (NT), mast cell conditioned (CM) medium or medium conditioned by mast cells treated with OGT2115 (2 µM for 24 hours; CM + OGT) and analyzed by RT PCR to evaluate *Muc1* (**A**; *n* = 9, Friedman’s *p* value: 0.256, contrasts *p* values are shown in the graph), estrogen receptor (*Esr1*; B, left panel; *n* = 9, Friedman’s *p* value: 0.001, contrasts *p* values are shown in the graph) and progesterone receptor expression (*Pgr*; **B**, right panel; *n* = 8, Friedman’s p-value: 0.002, contrasts *p* values are shown in the graph). **C** Expression levels of *Sox2* in PyMT41c (*n* = 9) cells treated as in panel A (Friedman’s *p*-value: 0.013, contrasts p-values are shown in the graph). Real Time PCR to evaluate *ESR1* (**D**; n = 3, Kruskal Wallis’s p-value: 0.010, Wilcoxon’s p-values are shown in the graph) and *PGR* (**D**; *n* = 6, Friedman’s *p* value: <0.001; contrasts p-values are shown in the graph), and MUC1 (**E**; *n* = 3, Kruskal Wallis’s p-value: 0.026, Wilcoxon’s *p* values are shown in the graph) expression in MCF7 cells cultured cultured as described above. **F** Western blot of MCF7 cells treated as indicated in (**D**), to evaluate the levels of MUC1-C. **G** Real time PCR to evaluate the expression level of estrogen receptor (*Esr1*; left panel; *n* = 4, interaction *p* value: 0.029, *p* value silencing in CM: 0.003, *p* value CM in siCtr: 0.001) and progesterone receptor (*Pgr*; right panel; *n* = 4, Friedman’s p-value is indicated) in PyMT41c cells transfected with a control silencing (siCtr) or with a siRNA directed against Muc1 (siMUC1) and cocultured or not with mast cells.

We first verified whether cancer cell treatment with mast cell conditioned medium was able to increase the expression levels of Muc1 in spheres (Fig. 3A), finding a modest increase partially prevented by OGT2115. We then asked if estrogen receptor expression was stimulated by mast cell conditioned medium in a heparanase-dependent manner and checked whether the estrogen receptor was active by evaluating the levels of its target gene progesterone receptor. Both transcripts were up-regulated upon conditioned medium stimulation, and heparanase inhibition could reduce estrogen receptor expression (Fig. 3B). We also tested whether the stem-associated genes were regulated in the same way and confirmed that *Sox2*, *Oct4* and *Nanog* were all up-regulated in spheres stimulated with mast cell conditioned medium (Fig. 3C and Supplementary Fig. 2), although heparanase inhibition could impair only *Sox2* increase (Fig. 3C), having no effect on Oct4 and Nanog expression (Supplementary Fig. 2). Findings were also verified with the human MCF7 cell line which confirmed that the inhibition of heparanase prevented mast cell-dependent *ESR1* and *PGR* up-regulation (Fig. 3D). Also in this case, mast cell conditioned medium failed to significantly upregulate *MUC1* transcription expression in MCF7 cells (Fig. 3E). Nonetheless, we evaluated MUC1 at protein level and found an increase of its MUC1-C active form, which was hindered via inhibition of heparanase (Fig. 3F). To test our idea that MUC1 could represent the link between mast cells and cancer cell estrogen receptor up-regulation, we silenced *Muc1* expression in PyMT41c cells (Supplementary Fig. S3) which were then co-cultured together with mast cells. As hypothesized, the presence of mast cells stimulated the expression of *Esr1*, together with its target gene *Pgr*, but the up-regulation was prevented by Muc1 silencing (Fig. 3G). These findings support the notion that mast cells stimulate estrogen receptor expression and activity, likely by increasing MUC1 activity.

### MUC1/ER axis promotes stem-like features of luminal breast cancer cells

**Fig. 4:**
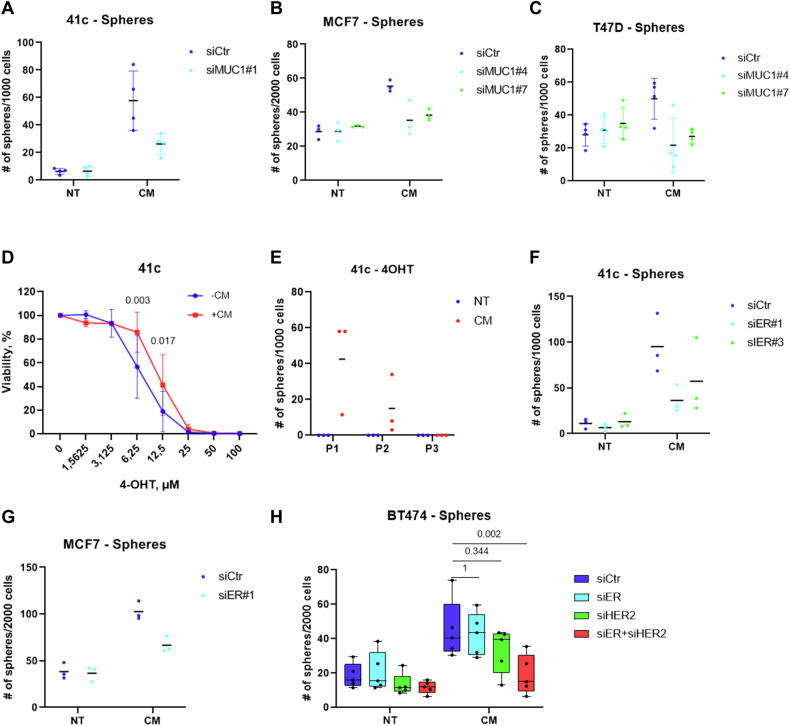
Mast cells promote stem-like features in luminal breast cancer cells through the MUC1/ER axis. **A** PyMT41c, (**B)** MCF7 and (**C**) T47D were silenced for MUC1 (siMUC1) or transfected with a nontargeting siRNA (siCtr), and tested in mammosphere forming assays in the presence of normal (NT) and mast cell conditioned (CM) medium. Spheres were counted after 7 days of culturing. In the PyMT 41c experiment, a silencing effect was observed in the CM group (*n* = 4, interaction p-value: 0.013; p-value silencing in CM: 0.002) as well as with MCF7 (*n* = 3, interaction p-value: 0.001; p-value silencing in CM: <0.001; *p* value CM in siCtr: <0.001) and T47D (*n* = 4, interaction p-value: <.0001; *p* value silencing in CM < .0001) cells. **D** Viability of PyMT41c cells treated with the indicated concentrations of tamoxifen for 72 hours, and cultured in mast cell conditioned medium (CM) or normal medium (NT) (interaction *p* value: 0.047, contrasts *p* values are shown in the graph). **E** Number of mammospheres formed by PyMT41c cells treated or not with tamoxifen (10 µM) and cultured with normal (NT) or mast cell conditioned (CM) medium (*n* = 3, interaction *p*-value: 0.028; p-value CM in P1: 0.004). **F** Mammosphere assay of PyMT41C and (**G**) MCF7 cells transfected with a siRNA specific for estrogen receptor (siER) or with a nontargeting siRNA (siCtr), and cultured in mast cell conditioned (CM) or normal (NT) medium. A silencing effect was observed in the CM group (PyMT41c: *n* = 3, interaction *p* value: 0.050; p-value silencing in CM: 0.005; MCF7: *n* = 3, interaction p- value: 0.023; *p* value silencing in CM: 0.004). **H** Number of spheres formed by BT474 cells transfected with a control silencing (siCTR), a siRNA specific for estrogen receptor (siER) or HER2 (siHER2), or the combination (siER + siHER2), and cultured in normal (NT) or mast cell conditioned medium (CM); (Friedman’s *p* value: <0.001; contrasts p-values are shown in the graph).

Having shown that ER is stimulated by mast cells in a MUC1-dependent manner, we asked whether this axis plays a role in the increased stem-like properties of mammary cancer cells. Hence, MUC1 was silenced in mouse (PyMT41c) and human (MCF7 and T47D) mammary cancer cells, and spheroids were counted in the presence of a medium conditioned by mast cells. In all models, we observed that MUC1 silencing caused a reduction of mammosphere forming capacity (Fig. 4A-C). Of note, MUC1 appears to be dispensable in this setting for HER2-positive cancer cells since MUC1 silencing has no effect on BT474 and TUBO cell capacity to form spheroids (Supplementary Fig. 4). Since we show here that the conditioned medium of mast cells stimulate the expression and activity of estrogen receptor, we asked whether the endocrine pathway was affected. In agreement with our hypothesis, we found that conditioned medium was sufficient to enhance the resistance to tamoxifen treatment in vitro (Fig. 4D). Also the capacity to form spheroids upon treatment with tamoxifen was enhanced in the presence of mast cell conditioned medium (Fig. 4E). Finally, we determined the role of estrogen receptor in the observed increase of stem-like properties by silencing ESR1 in breast cancer cells. In both PyMT41c and MCF7 cancer cells, the down-regulation of estrogen receptor caused a reduction of spheroids formed upon stimulation with mast cell conditioned medium (Fig. 4F, G). To further investigate the role of estrogen receptor in this process, we repeated the experiments with a number of both human (BT20, BT549) and mouse (E0771) triple-negative breast cancer cell lines, negative for estrogen receptor expression. In all the lines tested, the conditioned medium failed to increase the number of spheroids (Supplementary Fig. 5), supporting the notion that estrogen receptor is crucial for cancer cell stem-like properties induced by mast cell conditioned medium. Notably, also *ESR1* silencing had no effect on the capability of HER2-positive BT474 cancer cells to form spheroids further confirming that MUC1/ER axis is dispensable in this type of tumor (Supplementary Figure 6). Since BT474 cells express high levels of HER2, we hypothesize that this receptor could contribute to stemness making the estrogen receptor dispensable. Hence, we silenced estrogen receptor and/or HER2 in BT474 cells which were tested in mammosphere forming assay. We found that estrogen receptor or HER2 silencing alone were not sufficient to decrease the spheroid-forming capability of cancer cells, but the combined silencing significantly reduced the number of spheroids upon conditioned medium stimulation (Fig. 4H). This evidence supports the hypothesis that MUC1/ER axis plays a crucial role in conditioned medium-induced stemness induction in luminal HER2 negative cells, while HER2 expression can by-pass this axis. “Intrinsic” capability of cancer cells to form spheroids, i.e. not stemming from conditioned medium stimulation, is not affected by ER silencing.

### Mast cell heparan sulfate/TLR4 axis is crucial to trigger cancer cell stem-like properties

**Fig. 5:**
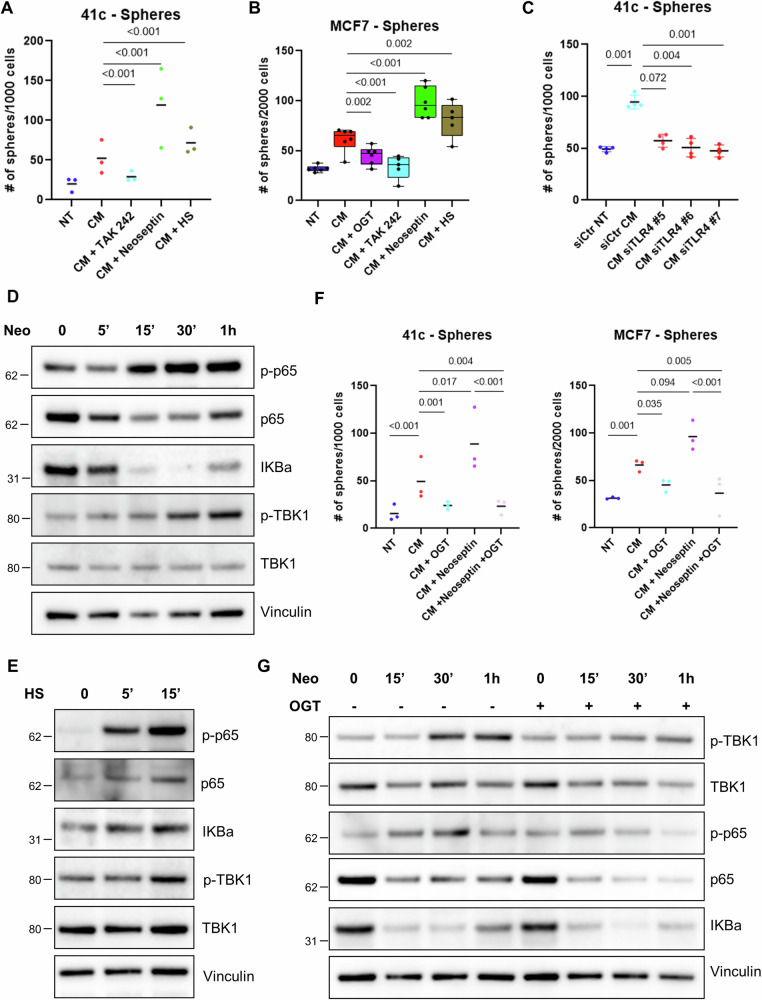
TLR4 stimulation promotes cancer cell stem-like properties. **A** PyMT41c cells (*n* = 3) and (**B**) MCF7 (*n* = 6) were tested in mammosphere forming assay by culturing in normal medium (NT), in medium conditioned by mast cells left untreated (CM), or treated for 24 hours with heparanase inhibitor (CM + OGT2115), TLR4 inhibitor (CM + TAK242), TLR4 agonist (CM + Neoseptin-3) and heparan sulfate (CM + HS). Friedman’s contrasts are shown. Spheres were counted after 7 days of culturing. **C** PyMT41c were tested in mammosphere forming assays in the presence of normal (NT) and mast cell conditioned (CM) medium collected from mast cells silenced with a control (siCtr) and three TLR4-specific (siTRL4) siRNAs, Friedman’s p-value: 0.003, contrasts are shown. **D** Mast cell protein lysates were collected after the treatment with TLR4 agonist (Neoseptin-3, 25 µM) or (**E**) with heparan sulfate (20 µg/ml) for the indicated time, and tested by western blot to evaluate the levels of phosphorylated and total p65 and TBK1, and IKBα. Vinculin is shown as loading control. **F** Mammosphere forming assay of PyMT41c (*n* = 3) and MCF7 (*n* = 3) cells cultured in the presence of normal (NT), mast cell-conditioned (CM) medium or medium conditioned by mast cells treated with heparanase inhibitor (CM + OGT2115), TLR4 agonist (CM + Neoseptin-3) or a combination of them (CM + Neoseptin-3 + OGT2115). Friedman’s *p*-value contrasts are shown. **G** Western blot of mast cells treated with TLR4 agonist for the indicated time and pre-treated or not with heparanase inhibitor for 3 hours to evaluate the level of phosphorylated and total TBK1 and p65, and IKBα. Vinculin is shown as loading control.

Mast cell heparanase activity is essential for estrogen receptor up-regulation and stem-like properties of cancer cells. Since it has been reported that cleaved heparan sulfate can stimulate macrophages via TLR4 [28], we asked whether this was valid also in mast cells. Moreover, we sought to understand if stimulation of TLR4 by cleaved heparan sulfate was responsible for our findings related to stem-like phenotype. To this end, we cultured PyMT41c cells with the conditioned medium collected from mast cells untreated, treated with heparanase inhibitor, with heparan sulfate and with TLR4 agonist and inhibitor. As hypothesized, heparanase (Fig. 2E) and TLR4 inhibition resulted in reduced capacity of cancer cells to form spheroids, although heparan sulfate itself and TLR4 agonist increased this effect (Fig. 5A). Similar results were confirmed by employing MCF7 cells (Fig. 5B) and by depleting mast cell TLR4 by silencing (Fig. 5C). Simultaneously, we investigated the effect of TLR4 stimulation on mast cells confirming the activation of the downstream pathways TBK1 and NF-kB upon TLR4 agonist and heparan sulfate administration (Fig. 5D, E, respectively). To further test whether heparan sulfate itself is responsible for the activation of the pathway, we combined heparanase inhibition with TLR4 agonist. Our hypothesis was that direct stimulation of TLR4 could by-pass the absence of heparan sulfate caused by heparanase inhibition. According to our speculation, TLR4 activation with another agonist would have been sufficient to promote the stem-like features of cancer cells also in the presence of inactive mast cell heparanase activity. Unexpectedly, the inhibition of heparanase blocked the whole process, strongly supporting the idea that functional heparanase is crucial for activation of TLR4 and heparan sulfate is not sufficient per se to produce the observed effects on cancer cell stemness (Fig. 5F). Accordingly, functional heparanase activity was found to be essential to completely activate the TLR4 pathway upon specific stimulation with Neoseptin-3 (Fig. 5G). Therefore, both heparan sulfate and TLR4 activation favor cancer stem-like properties, but active heparanase is essential for the whole process.

### Mast cell-dependent stimulation of cancer stem-like features rely on TNF

**Fig. 6:**
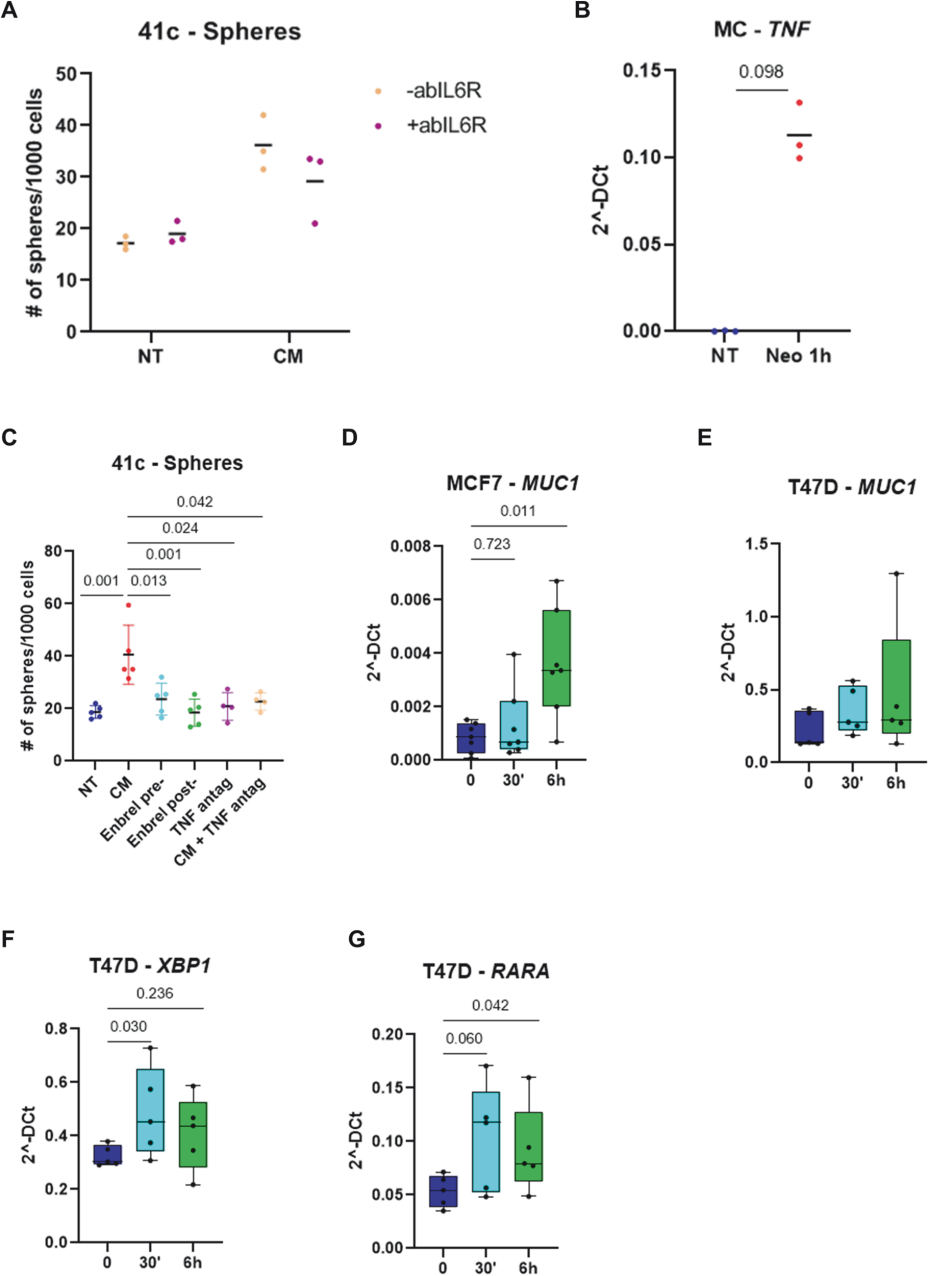
TNF is crucial for the induction of stem-related features by mast cells. **A** Mammosphere forming assay of PyMT41c cells cultured or not with an IL6 receptor neutralizing antibody (abIL6R) in the presence of normal (NT) or mast cell-conditioned medium (CM)(CM p-value: 0.001; abIL6R p-value: 0.375). **B** Real-time PCR to evaluate the expression levels of TNF of mast cells untreated or treated with TLR4 antagonist for 1 hour (Neo 1 h) Wilcoxon’s *p* value. **C** Number of spheroids formed by PyMT41c and MCF7 cells upon treatment with Enbrel or a TNF antagonist. Enbrel was used both before (Enbrel pre-) and after (Enbrel post) the collection of the conditioned medium from mast cells, while TNF antagonist treatment was made both on cells cultured with normal (NT + TNF antagonist) or mast cell-conditioned medium (CM + TNF antagonist). Friedman’s contrasts *p* values are shown. Real time PCR to evaluate the expression of MUC1 in MCF7 (**D**, Friedman’s p-value: 0.022) and T47D (**E**, Friedman’s p-value: 0.178) cells and of XBP1 (**F**, Friedman’s p-value: 0.085) and RARA (**G**, Friedman’s *p* value: 0.078) in T47D upon treatment with TNF (50 ng/ml) for 30’ and 6 hours. Friedman’s contrasts p-values are shown in each graph.

Having shown that mast cell conditioned medium is sufficient to promote the capability of mammary cancer cells to form spheroids, we looked for cytokines that could contribute to this effect. Since IL6 has been shown to promote EMT and stem potential in both normal and cancer cells, we added its inhibitor in the medium before stimulating the cancer cells. However, IL6 blocking did not affect the spheroid-promoting effect of the conditioned medium (Fig. 6A). We then investigated the effect of TNF, whose expression is triggered by TLR4 stimulation (Fig. 6B) and confirmed that its blocking with either Enbrel or a TNF antagonist could prevent the capability of the conditioned medium to promote spheroid formation (Fig. 6C). We hence concluded that mast cell-derived TNF and/or cancer cell TNF triggered by mast cell-derived soluble factors is essential to stimulate the MUC1/estrogen receptor axis and promote the stem-like properties, eventually increasing the aggressiveness of mammary cancer cells.

To verify whether TNF could be responsible for the activation of the MUC1/estrogen receptor pathway, we directly stimulated T47D and MCF7 cells with TNF finding, that this cytokine indeed has the potential to stimulate MUC1 (Fig. 6D-E), but, more importantly, it triggers the estrogen receptor activity. In fact, RARA and XBP1, two estrogen receptor target genes were both up-regulated by TNF administration (Fig. 6F, G), confirming a putative role of this cytokine in the pathway investigated in our work.

## Discussion

Mast cells, which have been widely neglected in the past, are now attracting more and more interest in the oncology field. In fact, although there are relatively few mast cells infiltrating tumors, they are endowed with prognostic and predictive value [2]. The advent of modern technology allowing single cell analysis and the exploiting of genes specifically expressed by mast cells [9, 29] now permit to pinpoint the presence and the density of these immune cells in the tumor microenvironment.

Mast cells are associated with both positive and negative prognostic value although the underlying mechanisms are still poorly characterized. Different types of tumors, and diverse subtypes of the same tumors, are characterized by variable quantity of mast cell infiltration [5]. For example, luminal, basal and HER2-positive breast cancers are all characterized by different amounts of infiltrating mast cells [5, 30]. Moreover, although infiltrating mast cells are linked to better prognosis in basal breast cancer, they display an opposite role in luminal breast cancer. Notably, a different effect of mast cells was observed also in our work, where stem-like properties are induced by mast cells only in the presence of luminal, but not triple negative/basal breast cancer cells. Mast cells have been shown to promote an immunosuppressive tumor microenvironment [14], to hinder the efficacy of therapies [11, 31] and favor cancer cell features associated to increased aggressiveness. On the contrary, in a few tumor types, they are known to stimulate an anti-tumor immune response [13, 32] and to prevent a phenotype of cancer cells associated with worse prognosis. In our work, we show that mast cells favor tumor-initiating potential of cancer cells, but that the mechanisms are different according to the presence of HER2 expression. The comprehension of the soluble factors involved in this process could contribute to the understanding of why mast cells could be endowed with diverse potential in different cancer settings. In relation to this, we demonstrate here that a crucial role could be played by heparanase, which is the only enzyme able to cut heparan sulfate proteoglycans [21]. In this way, heparanase profoundly shapes the tumor microenvironment affecting several cancer cell features. Its activity allows the modification of extracellular matrix and the release of cytokines and chemokines, which can exert their function and increase their activity. This is the case for example of FGF2 [33], a ligand known to promote cancer cell stemness [34]. Having confirmed that heparanase is essential for mast cell capability to promote cancer cell stem-like features, we hence hypothesized that heparan sulfate could be responsible for the observed effect by TLR4 activation. Our findings support this notion since both heparan sulfate and a TLR4 agonist could both result in the same effect, and TLR4 inhibitors showed an opposite outcome. Nonetheless, the inhibition of heparanase demonstrated that heparan sulfate alone, nor TLR4 stimulation, is not sufficient to favor cancer cell stem-like properties and an active heparanase is always essential. Although we have not clarified this aspect, the reason for this observation could stem from the fact that heparanase regulates a plethora of mechanisms both upstream and downstream TLR4 activation. Heparanase, for example, could be necessary for TLR4 activation via heparan sulfate release, but also crucial for a complete secretion of cytokines or mast cell functions, such as degranulation. In view of this, a role could be played by Syndecan-4, a heparan sulfate proteoglycan, which is necessary for heparanase activity and mast cell degranulation [35], and could hence represent a link between these two events.

In our work, we show that cancer cell MUC1 is crucial for estrogen receptor expression and activity, and for the increased capacity to form spheroids. Although MUC1 transcriptional levels were modestly increased upon conditioned medium stimulation, nonetheless, the active MUC1-C protein was upregulated. Moreover, the silencing of total MUC1 could prevent both estrogen receptor expression and spheroid formation stimulated by mast cells. This observation supports the idea that MUC1 is crucial in this pathway without being transcriptionally stimulated as initially hypothesized.

The main limitation of our work is that, although the starting observation was made in vivo, successive work was performed in vitro and with ex vivo-generated mast cells. In fact, models to study cell-cell interactions are extremely difficult to be evaluated in vivo, also considering that soluble factors were shown to be crucial in our observations with conditioned medium, making even more complicated to investigate the molecular mechanisms involved. Differences are obviously present between the in vivo and in vitro settings. For example, we have found that Sox2, Nanog and Oct4 are all increased in vitro by mast cell conditioned medium, although only Sox2 appears to be regulated by the heparanase/Muc1/estrogen receptor axis since it was the only one inhibited by OGT2115 treatment. More work is required to dissect the underlying mechanism and the specificity of Sox2 regulation by heparanase. In contrast, only Oct4/Pou5f1 was found to be up-regulated by mast cells in vivo. This discrepancy could be due to the fact that different transcription factors are necessary for sustaining tumor initiating in in vivo and spheroid formation in vitro, or that Sox2 and Nanog are expressed also by other cells in the tumor microenvironment, hence “contaminating” gene expression analysis. Moreover, in vitro we were able to employ mast cell conditioned medium, whereas in vivo mast cells were co-injected with cancer cells.

In conclusion, with our work, we describe for the first time that mast cells have the potential to stimulate and promote the tumor-initiating potential of cancer cells, also providing the underlying molecular mechanisms. More experiments are necessary to identify the soluble factors released by mast cells and identify the inhibitors to counteract this effect in tumors. Nevertheless, our data further support the idea that mast cells could represent a target in tumor therapy and describe a possible reason to explain why these immune cells display different effects based on the tumor type investigated. In fact, although we show that mast cells stimulate stem-like properties in both HER2-positive and –negative tumors, they rely on estrogen receptor activity only in the latter settings. In combination with treatment, mast cell prognostic value could hence be different according to the type of therapy.

## Material and methods

### Cell culture and treatments

The mouse mammary PyMT41C cell line was established in our laboratory from spontaneous tumors collected from MMTV-PyMT B6 mice as previously described [5]. BT474, BT20, T47D, BT549 and MCF7 cell lines were purchased from ATCC. Cells were cultured in DMEM (Gibco-Thermo Fisher Scientific) supplemented with 10% fetal bovine serum (FBS) (Euroclone), 2 mM L-glutamine (cat. no. 56-85-9; Merck), 1 mM sodium pyruvate (cat. no. 13-115E; Lonza), and 1× non-essential amino acid solution (cat. no. M7145, Merck). Cell lines were tested once a month for mycoplasma by using the PCR Mycoplasma Detection Set (cat. no. 6601, Takara). Cells were treated with 2 µM heparanase inhibitor OGT2115 (cat. no.2710; Tocris Bioscience), 1 µM TLR4 inhibitor TAK-242 (cat. no. 243984-11-4; Calbiochem), 25 µM TLR4 agonist Neoseptin-3 (cat. no. SML1686; Merck), 20 µg/ml heparan sulfate (cat. no. H7640; Merck), 10 µM 4-Hydroxytamoxifen (cat. no. H6278; Merck), 50 µg/ml TNF inhibitor enbrel (etanercept; Wyeth Pharmaceuticals), 10 µM TNF antagonist (cat. no. 654255; Merck), TNF ligand [36] and 50 ng/ml IL6 receptor neutralizing antibody [37].

### Animals and treatments

C57BL/6 ^W-sh/W-sh^ (Wsh) mice were purchased from The Jackson Laboratory, and maintained under pathogen-free conditions at the animal facility of Fondazione IRCCS Istituto Nazionale dei Tumori (Milan, Italy). Experiments were approved by the Ethics Committee for Animal Experimentation of the Fondazione IRCCS Istituto Nazionale dei Tumori of Milan and by the Italian Ministry of Health (authorization number 1205/2020-PR). Female mice were injected with 2.5×10^3^, 2.5x10^4^ and 2.5x10^5^ PyMT41c with or without the same number of bone marrow-derived mast cells and monitored twice a week for tumor growth. Tumor volume was measured with the following formula: V = D x d^2^/2, where D and d represent the major and minor diameter, respectively. Mice were considered engrafted when tumor reached the size of 3x3 mm.

### Bone marrow-derived mast cells and conditioned medium

Bone marrow was harvested from the femurs of female C57BL/6 mice of about 6 weeks of age. Femurs were dissected to remove muscle fragments, and one extremity was cut with sterile scissors. Bone marrow cells were flushed out by inserting in the medullar canal the needle of a 5 mL syringe filled with sterile phosphate-buffered saline. The pooled cell suspension was cultured in vitro in RPMI 1640 medium (Gibco-Thermo Fisher Scientific) supplemented with 20% FBS, 2 mM L-glutamine, 1 mM sodium pyruvate, and 1× non-essential amino acid solution, 20 ng/ml stem cell factor (cat. no. AF-250-03; PeproTech) and 20 ng/ml interleukin-3 (cat. no. 213-13; PeproTech). Once a week, non-adherent cells were centrifuged, resuspended in fresh culture medium, and placed in new culture flasks. After 4 weeks, the purity of bone marrow-derived mast cells was evaluated by measuring the percentage of FceRI- and cKit-positive and CD11b negative cells on an LSRII Fortessa flow cytometer (BD Biosciences). The following antibodies were used: PE-cyanine7-conjugated anti-mouse CD117/cKit (cat. no. 25-1171-82; Thermo Fisher Scientific), FITC-conjugated anti-mouse FceRI (cat. no. 11-5898-85; Thermo Fisher Scientific) and APC-conjugated anti-mouse CD11b (cat. no. 17-0112-83; Thermo Fisher Scientific). Data were analyzed using FlowJo software. Mast cells were used for in vitro experiments only when culture purity was >90%.

To prepare mast cells conditioned medium, mast cells were seeded at 1×10^6^ cells/ml in culture media without any supplement. After 24 hours, the medium of these cultures was collected and mixed 1:1 with fresh media with a double concentration of all supplements. This resulting medium was used as conditioned medium to treat cells in subsequent experiments.

### Mammosphere forming assay

Single-cell suspensions of cells were plated in low-attachment, 6-well plates at a density of 1000 cells/ml, for PyMT41c, EO771, BT549 and T47D cells or 2000 cells/ml for BT474, BT20, E0771, TUBO and MCF7 cells in serum-free DMEM-F12 (Thermo Fisher Scientific) supplemented with 20 ng/ml epidermal growth factor (cat. no. 315-09; Peprotech), 20 ng/ml basal fibroblast grow factor (cat. no. 100-18; PeproTech), 5 μg/ml insulin (cat. no. 19278; Merck) and 0,4% BSA (cat. no. A4378; Merck). Spheres were counted after 7 days (first passage, P1) of culturing. Then, spheres were disaggregated and cells seeded in the same conditions as P1 to evaluate the number of spheroids after 7 days (P2), and then again after further 7 days upon disaggregation (P3). In each passage, spheres were disaggregated using TripLE express (cat. no. 12604-013; Thermo Fisher Scientific) and seeded again in the same condition as P1. Mammospheres were imaged using an EVOS XL core digital inverted microscope (Thermo Fisher Scientific) with 10× magnification.

### Lentiviral transduction

To check the expression of Sox2 upon the treatment with mast cell conditioned medium, we obtained PyMT41c cells stably expressing a GFP-based Sox2 promoter reporter. Cells were transduced with lentiviral particles obtained from HEK293 cells transfected with pGreenFire1-Sox2SRR2-mCMV-EF1-Puro (cat. no. SR20071-PA-P; System Biosciences) as already described [38]. After 72 h, transduced cells were selected using 1 μg/ml Puromycin (cat. no. A11138-03; Thermo Fisher Scientific).

### RNA extraction and real-time PCR

Total RNA of spheres obtained from PyMT41c cells was extracted at the end of P1 with Quick-RNA MicroPrep Kit (Zymo) while total RNA from MCF7 cells was obtained with miRNeasy Mini Columns (Qiagen) according to the manufacturer’s instructions. RNA (1 μg) was reverse-transcribed using the High Capacity cDNA Reverse Transcription Kit (Thermo Fisher Scientific), and real-time PCR was carried out with TaqMan Fast Universal PCR Master Mix (Thermo Fisher Scientific). Probes for *Sox2* (Mm03053810_s1), *Esr1* (Mm00433149_m1; Hs01046816_m1), *Pgr* (Mm00435628_m1; Hs01556702_m1), *Muc1* (Mm00449604_m1, Hs00159357_m1), *Pou5f1* (Mm03053917_g1) *Nanog* (Mm02019550_s1), TNF (Mm00443258_m1), Rara Hs00940446_m1; Xbp1 Hs00231936_m1, *Actb* (Mm00607939-s1) and *Gapdh* (Mm99999915_g1; Hs02786624_g1) were purchased from Thermo Fisher Scientific. Samples were analyzed using QuantStudio 3 software (Thermo Fisher Scientific), and transcript levels of technical duplicates were quantified using the Delta-Ct method and normalized to *Gapdh* expression for cancer cells and to *Actb* expression for mast cells. For this assay, MCF7 cells were cultured in DMEM (Gibco-Thermo Fisher Scientific) without phenol-red and supplemented with 0,5% charcoal stripped fetal bovine serum (cat. no. A33821-01; Gibco-Thermo Fisher Scientific), 2 mM L-glutamine, 1 mM sodium pyruvate, and 1× non-essential amino acid solution. For the analysis of transcript from ex vivo samples, mRNA was extracted from tumors collected after reaching 6x6 mm size from Wsh mice injected with 10^5^ PyMT41c with or without the same number of mast cells. Tissues were minced and dissociated with Collagenase/Hyaluronidase (cat. no. 07912; Stemcell technologies) for 2 hours at 37 °C according to manufacturer’s instructions.

### Heparanase ELISA

Heparanase levels were quantified in the medium conditioned for 24 hours by mast cells using mouse ELISA Kit for Heparanase (cat. no. SEA711Mu-96t; Cloud-clone corp) according to the manufacturer’s instructions.

### Gene silencing

To obtain transient silencing of target genes, cells were transfected with a reverse transfection protocol using Lipofectamine RNAiMAX transfection reagent (Thermo Fisher Scientific) with siRNA specific for ERα (cat. no. SI00996219 and SI00996233; Qiagen for the mouse cell line and cat. no. 4392420; Invitrogen for human cell lines), MUC1 (cat. no. SI00207004 for the mouse cell line, cat. no. SI00038143 and SI02780673 for the human cell line; Qiagen), HER2 (cat. no. SI0063882; Qiagen), TLR4 (cat. no. 188775, 188776 and 188777) and negative control siRNA (cat. no. 3510999, Qiagen) as control of transfection.

### Western Blot

Protein lysates were obtained resuspending pellets in lysis buffer (125 mM Tris HCl pH 6.8, 5% SDS) and boiling it for 10 minutes at 99 °C. Lysates were sonicated for 20 seconds and clarified by centrifugation at 13000 rpm for 15 minutes. The extraction and separation of cytoplasmic and nuclear protein fractions was made using NE-PER™ Nuclear and Cytoplasmic Extraction Reagents; cat. no. 78833 (Thermo Fisher Scientific) according to the manufacturer’s instructions. Proteins (30-50 μg) were separated by SDS-PAGE on precast 4%–12% Bis-Tris NuPAGE gels (Thermo Fisher Scientific) and transferred to PVDF membrane (Merck). Upon saturation for 30 minutes in Tris-buffered saline containing 4% BSA, membranes were incubated overnight with the following primary antibody: anti-mouse p-p65 (cat. no. 3033), anti-mouse p65 (cat. no. 6956), anti-mouse p-TBK1 (cat. no. 5483), anti-mouse TBK1 (cat. no. 3504), anti-mouse IKBα (cat. no. 9242), anti-mouse p-ERK1/2 (cat. no. 9101), anti-mouse ERK1/2 (cat. no. 9102), anti-mouse HDAC1 (cat. no. 34589), anti-human MUC1-C (cat. no. 16564), all from Cell Signaling Technology, and anti-actin (A1978; Merck), anti-vinculin (cat. no. V9131; Merck) and alpha Tubulin (cat. no. sc-32293; Santa Cruz Biotechnology). Proteins were detected upon hybridization with appropriate HRP-conjugated secondary antibodies by adding a chemiluminescent substrate (Takara) and acquired by using the Azure byosistem 600 (Aurogene). Original uncropped western blots are provided as supplemental material.

### Viability assay

PyMT41c cells were seeded in a white 96 well plate (1×10^4^ cells/well) and after 24 hours treated with 4-hydroxytamoxifen from 1 µM to 100 µM. After 72 hours, cell viability was evaluated according to manufacturer’s protocol, through Celltiter-Glo Luminescent Cell Viability assay (cat. no. G7571; Promega) and luminescence was measured using the acquisition instrument Spark (Tecan).

### Statistical analyses

Dotplots reporting means with Standard Deviations (SD) (if n > 3) or boxplots, according to the number of observations, were plotted in order to describe the data. To jointly consider observations obtained from experiments performed at different times, Friedman’s nonparametric ANOVA or the parametric ones were fitted according to the number of factors of interest. Differences between groups of interest were assessed with the nonparametric Wilcoxon (W) or Kruskal-Wallis (KW) exact tests by estimating p-values through the Monte Carlo approach to handle sample size limitations if any. The equality of two proportions obtained from independent samples was tested in terms of difference (Δp) by using the Pearson Chi-Square test and the corresponding 95% Confidence Interval (95% CI). A mixed-effects model was implemented on the viability data by considering the factor *date of experiment* as random and the levels of 4-OHT and CM as fixed, in order to evaluate their joint effect on the viability. All the above analyses were performed using SAS® Studio software (Release 5.2.; SAS Institute, Inc., Cary, NC, USA) by considering a significance level of alpha = 0.05.

## Supplementary information

Supplementary Figure 1

Supplementary Figure 2

Supplementary Figure 3

Supplementary Figure 4

Supplementary Figure 5

Supplementary Figure 6

Supplementary Figure Legends

Original Data

## Data Availability

Data sharing not applicable to this article as no datasets were generated or analysed during the current study.
