# Supplementary figures and images for: Mast cell heparanase promotes breast cancer stem-like features via MUC1/estrogen receptor axis
Source: Cell Death Dis. 2024 Sep 30;15(9):709. doi: 10.1038/s41419-024-07092-9 (PMC11442964; doi:10.1038/s41419-024-07092-9)

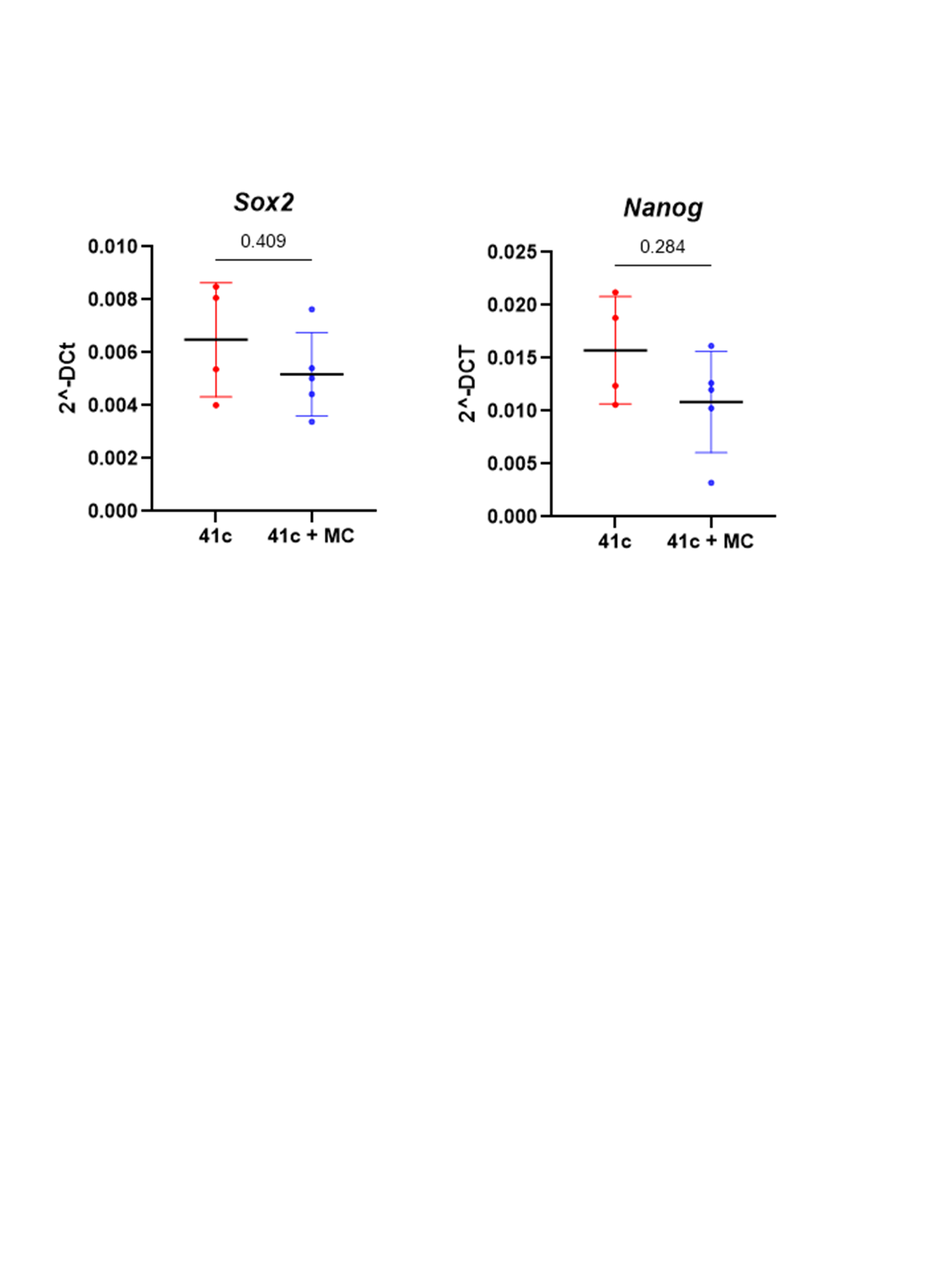

Supplement: Supplementary file 1 — Supplementary Figure 1 [file 41419_2024_7092_MOESM1_ESM.tif]

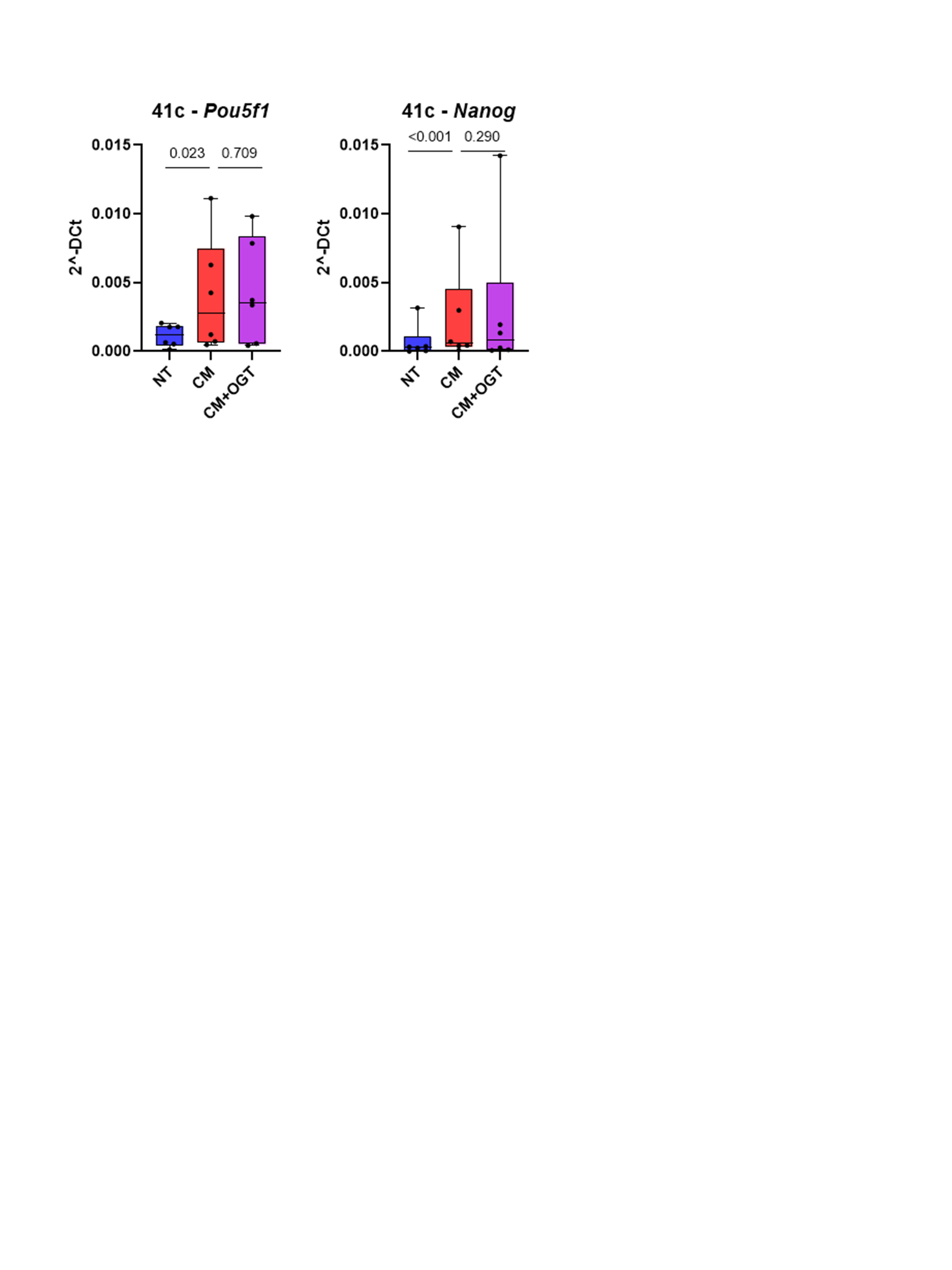

Supplement: Supplementary file 2 — Supplementary Figure 2 [file 41419_2024_7092_MOESM2_ESM.tif]

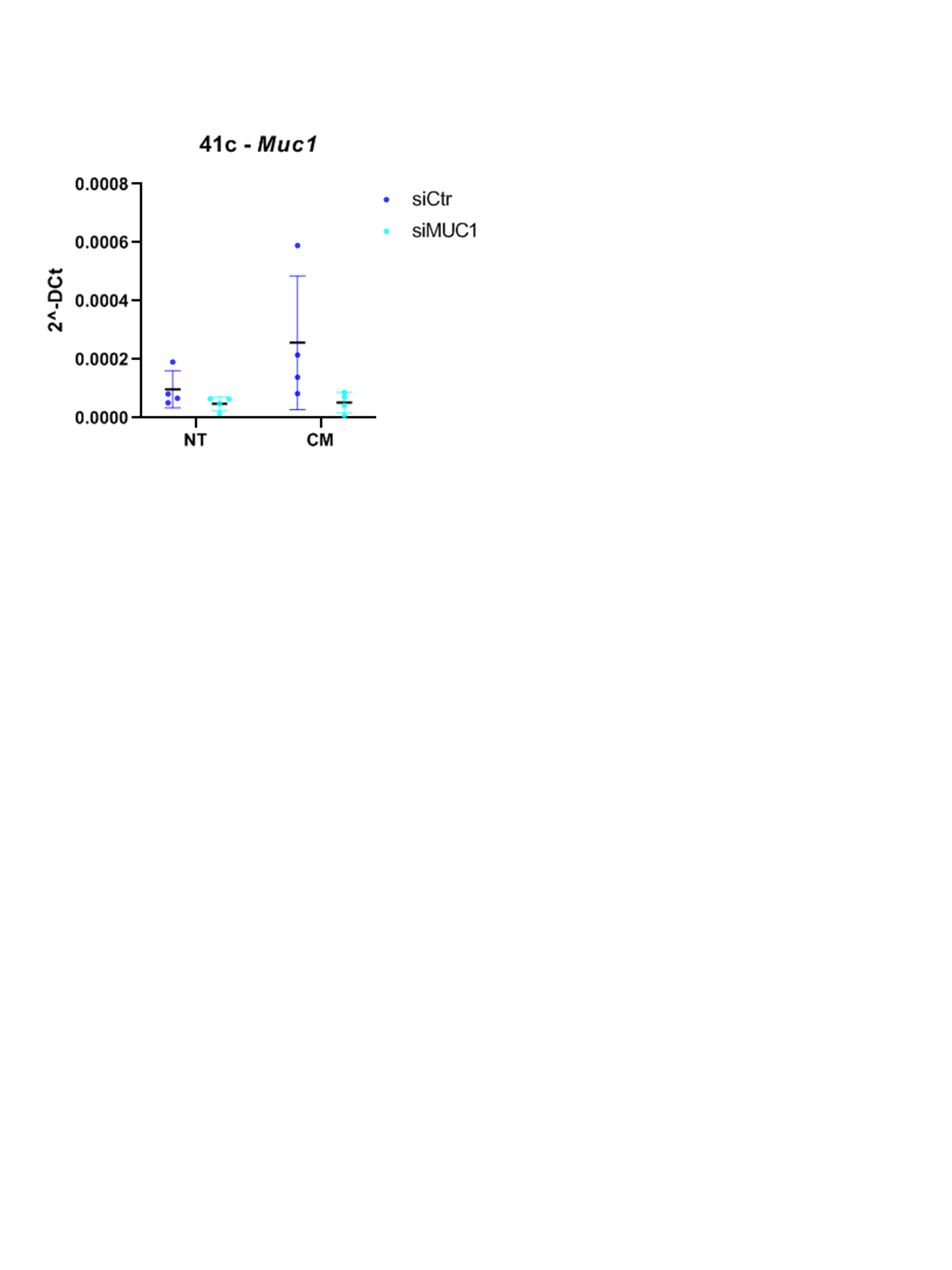

Supplement: Supplementary file 3 — Supplementary Figure 3 [file 41419_2024_7092_MOESM3_ESM.tif]

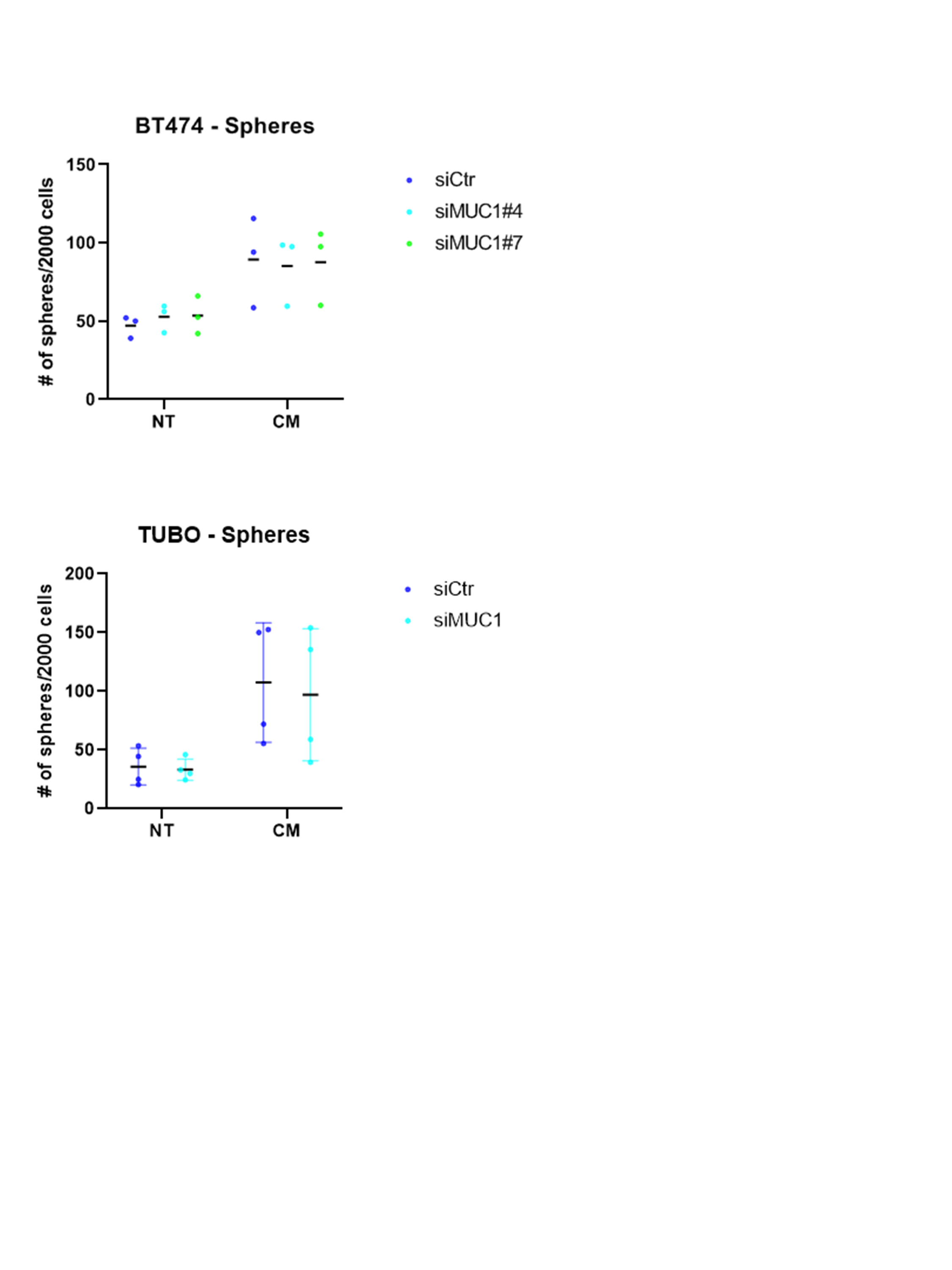

Supplement: Supplementary file 4 — Supplementary Figure 4 [file 41419_2024_7092_MOESM4_ESM.tif]

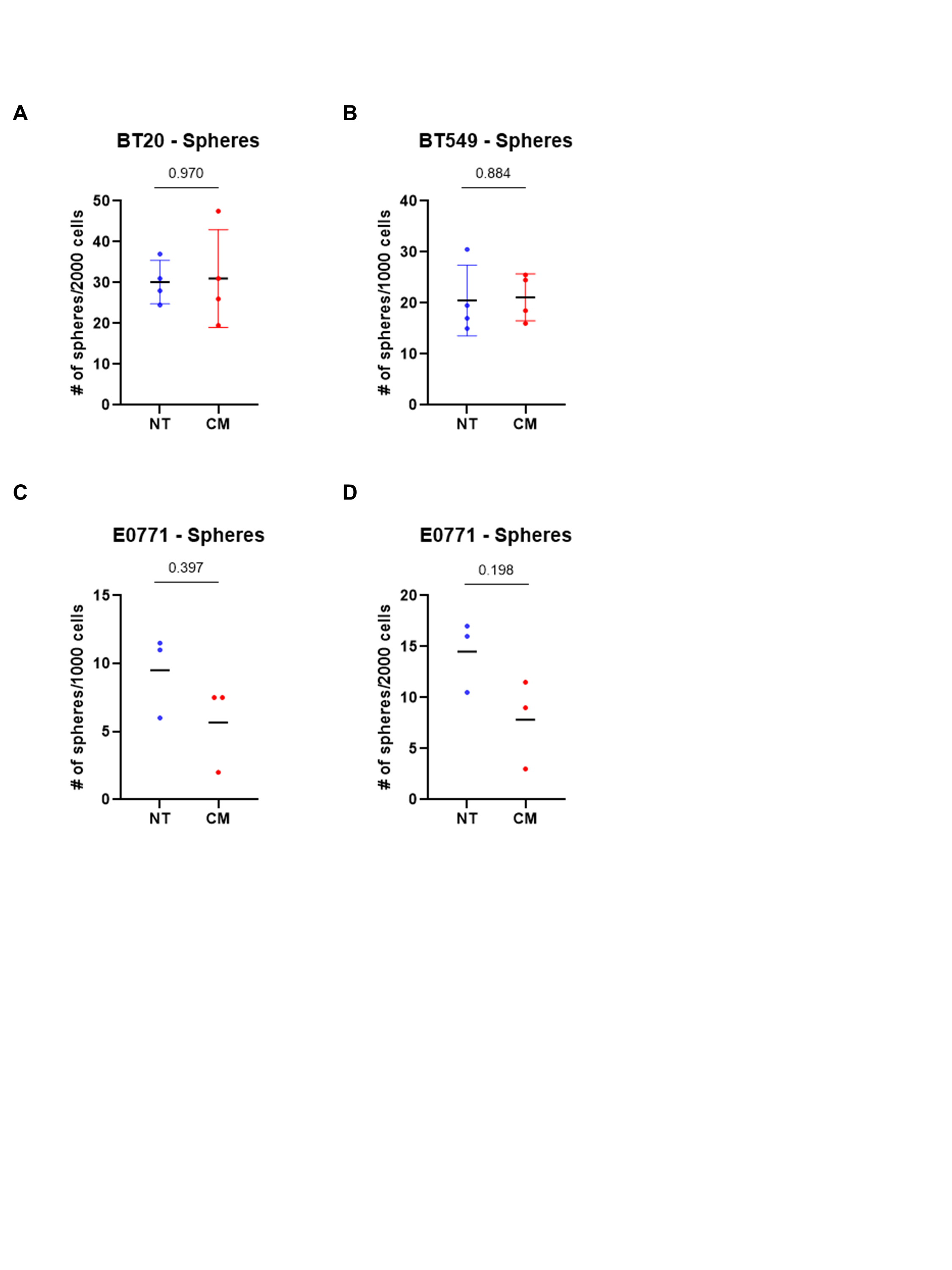

Supplement: Supplementary file 5 — Supplementary Figure 5 [file 41419_2024_7092_MOESM5_ESM.tif]

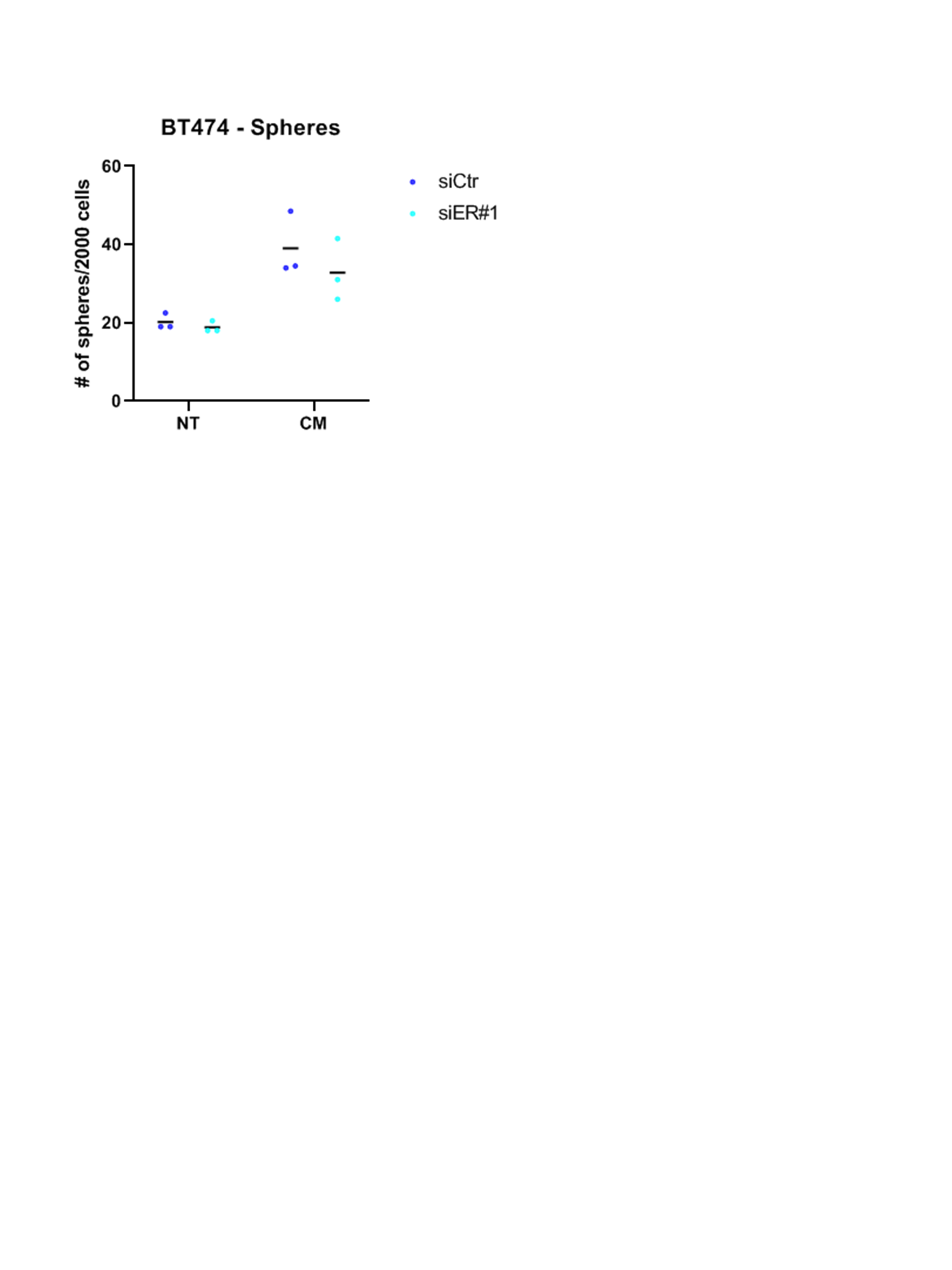

Supplement: Supplementary file 6 — Supplementary Figure 6 [file 41419_2024_7092_MOESM6_ESM.tif]

**Figure 2b**

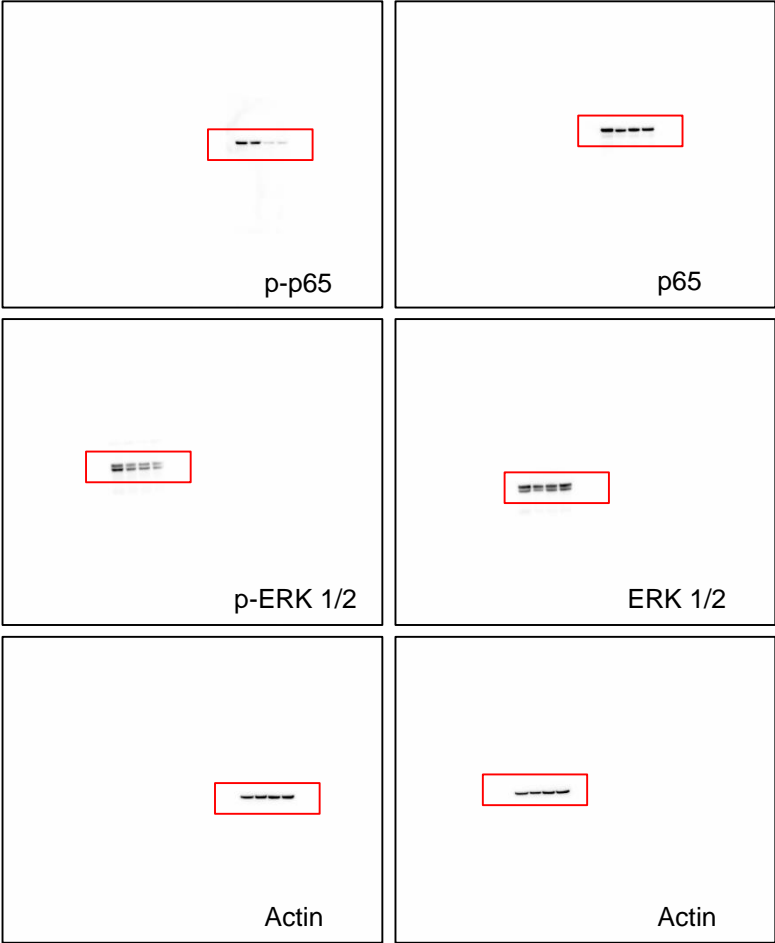

**Figure 2c**

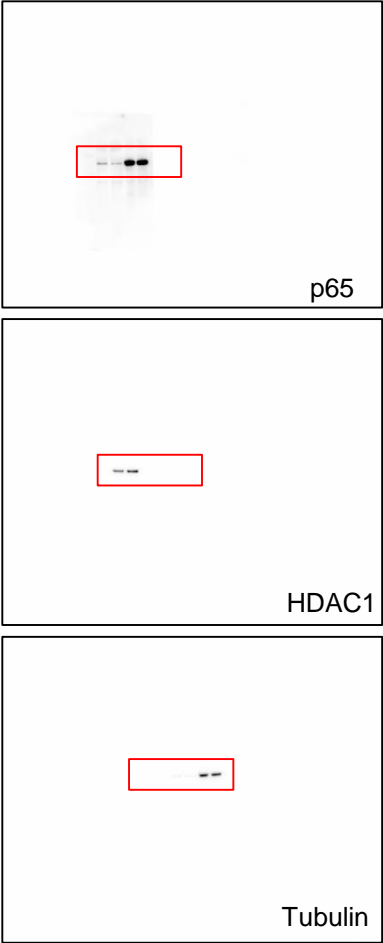

**Figure 3f**

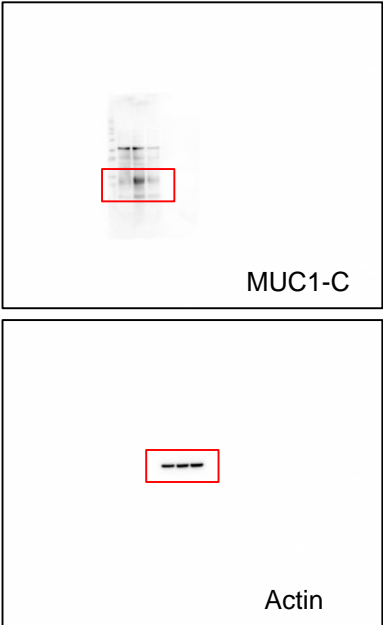

Figure 5c

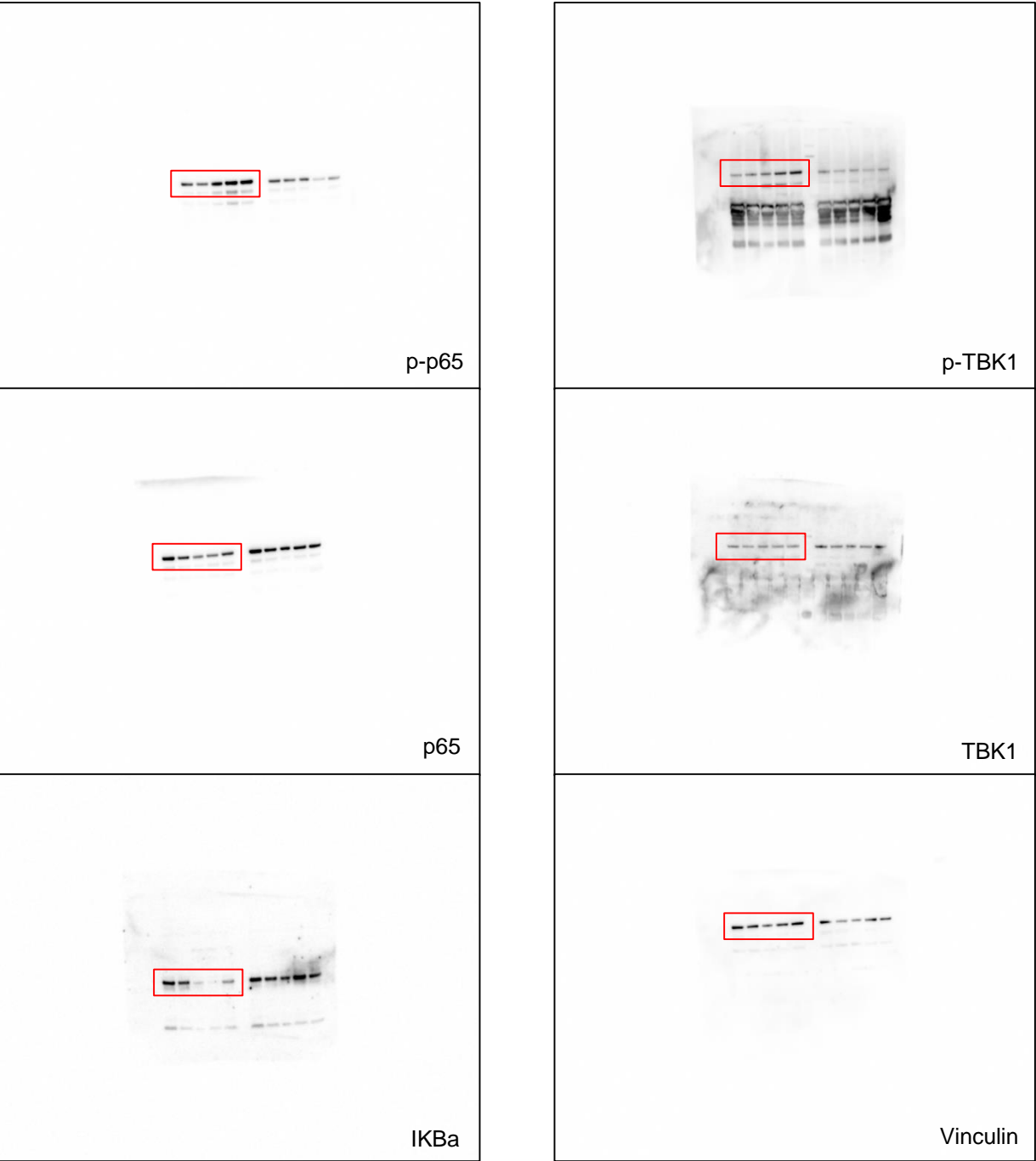

Figure 5d

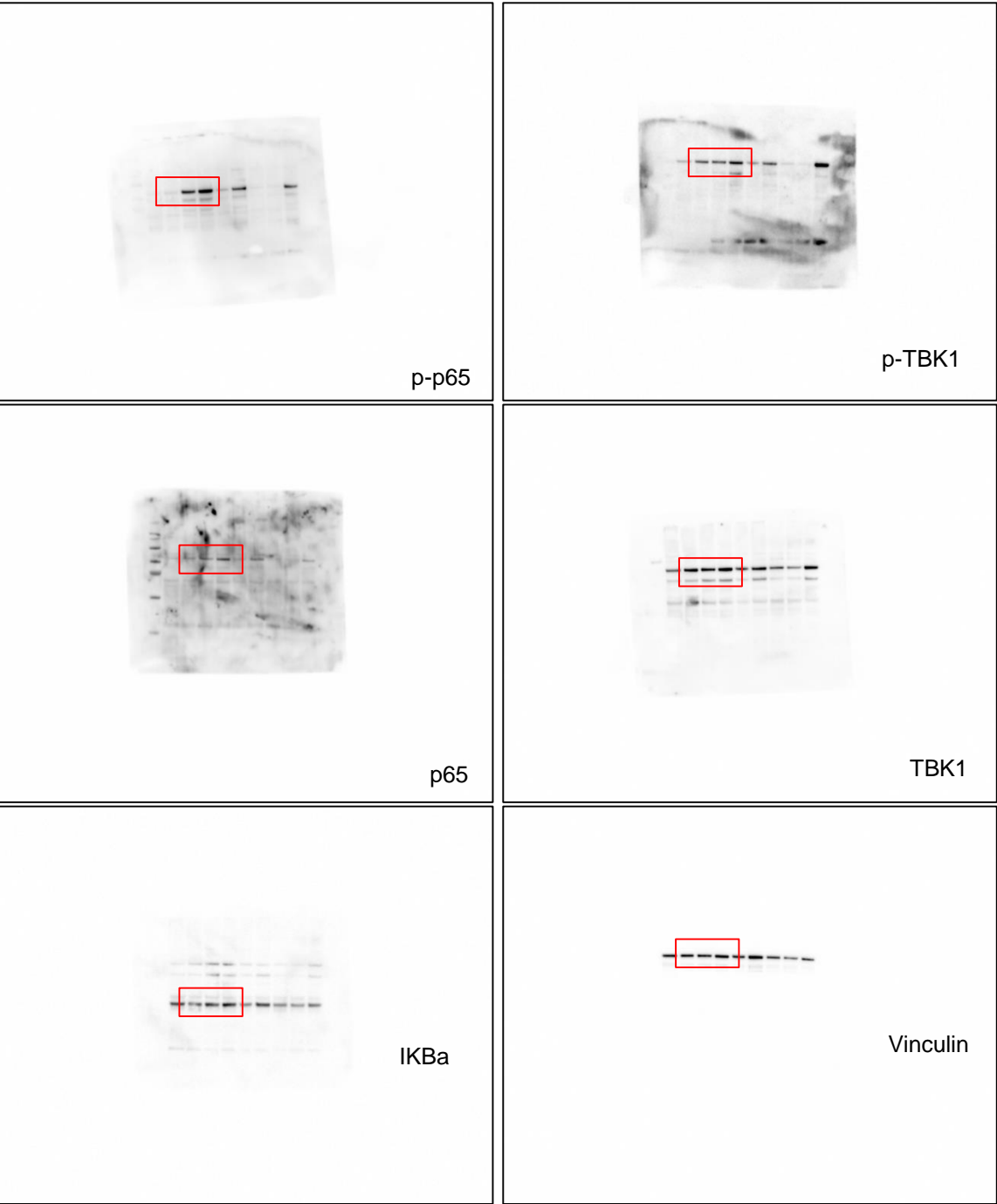

Figure 5f

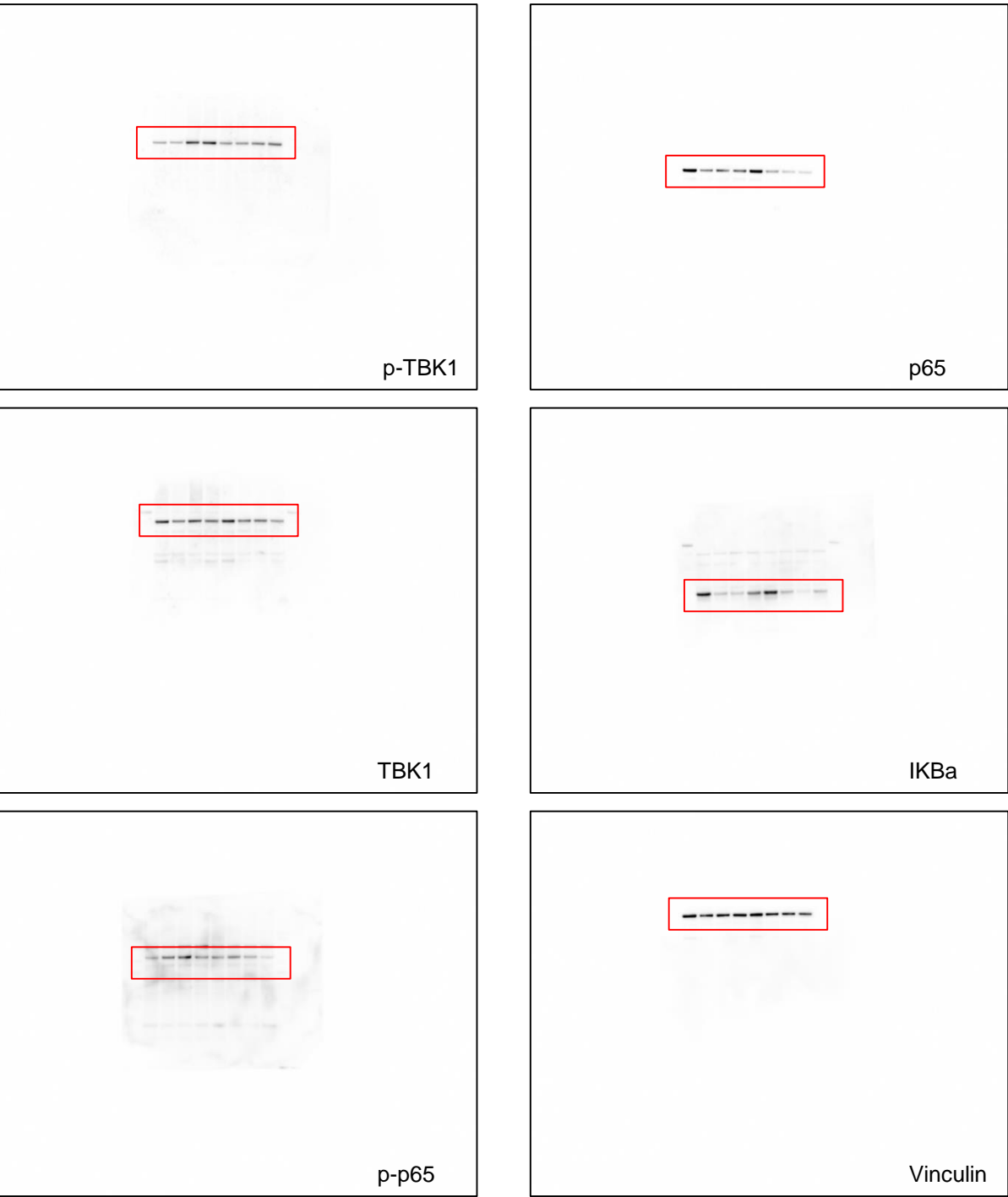

Supplement: Supplementary file 8 — Original Data [file 41419_2024_7092_MOESM8_ESM.pdf]
