# Supplementary material for: Mast cell heparanase promotes breast cancer stem-like features via MUC1/estrogen receptor axis
Source: Cell Death Dis. 2024 Sep 30;15(9):709. doi: 10.1038/s41419-024-07092-9 (PMC11442964; doi:10.1038/s41419-024-07092-9)
Supplement: Supplementary file 7 — Supplementary Figure Legends [file 41419_2024_7092_MOESM7_ESM.docx]

Supplementary Figure 1. Ex vivo analysis of Sox2 and Nanog mRNA levels expressed by tumors grown in the presence of mast cells (41c + MC, n = 5) or not (41c, n = 4). Wilcoxon’s p-value is shown.

Supplementary Figure 2. Real time PCR to evaluate the expression of Pou5f1 (n=6, Friedman’s p-value: 0.024) and Nanog (n=6, Friedman’s p-value: <0.001) in PyMT41c cells cultured in normal (NT), mast cell-conditioned (CM) medium or medium conditioned by mast cells treated with OGT2115 (2 µM for 24 hours; CM + OGT2115). Friedman’s contrasts p-values are shown in each graph.

Supplementary Figure 3. Real time PCR to verify the expression level of Muc1 (n=4) in PyMT41c cells transfected with a control silencing (siCtr) or with a siRNA directed against Muc1 (siMUC1), and cocultured or not with mast cells. (CM p-value: 0.393; silencing p-value: 0.014)

Supplementary Figure 4. Number of mammospheres formed by BT474 and TUBO silenced or not for MUC1, and cultured in normal (NT) or mast cell conditioned medium (CM) (BT474: CM p-value: <0.001; silencing p-value: 0.913; TUBO CM p-value: 0.001; silencing p-value: 0.679).

Supplementary Figure 5. Human (2000 BT20 cells/well, A, 2000 BT549 cells/well, B) and mouse (1000 and 2000 E0771 cells/well, C and D respectively) triple negative breast cancer cells were tested in mammosphere forming assay in the presence of normal (NT) and mast cell conditioned (CM) medium. Wilcoxon’s p-value is shown.

Supplementary Figure 6. Number of spheroids formed by BT474 cells in the presence of normal (NT) or mast cell conditioned medium (CM), upon transfection with a control silencing (siCtr) or a silencing directed against ER (siER); (CM p-value: 0.001; silencing p-value: 0.255).
